# Bioactive Phytochemicals from *Mercurialis* spp. Used in Traditional Spanish Medicine

**DOI:** 10.3390/plants8070193

**Published:** 2019-06-28

**Authors:** José Blanco-Salas, Francisco M. Vazquez, María P. Hortigón-Vinagre, Trinidad Ruiz-Tellez

**Affiliations:** 1 Department of Vegetal Biology, Ecology and Earth Science, Faculty of Sciences, University of Extremadura, 06071 Badajoz, Spain; 2 Department of Forest Production and Biodiversity, Institute of Research Agrarian Center La Orden—Valdesequera, Scientific and Technological Research Center of Extremadura (CICYTEX) A5 Km 372, 06187 Guadajira, Badajoz, Spain

**Keywords:** bioactive phytochemicals, *Mercurialis* spp., traditional Spanish medicine

## Abstract

Plants from the genus *Mercurialis* have a long history of use as herbal remedies in traditional Spanish medicine. The growing interest in the conservation of knowledge related to biodiversity has encouraged us to review the bioactive phytochemicals from the four most widespread *Mercurialis* species in the Iberian Peninsula (*M. annua* L., *M. ambigua* L., *M. perennis* L., and *M. tomentosa* L.). First, the medicinal uses of these four species throughout Spain were compiled, and then a bibliographical search on their chemical composition was conducted in an attempt to justify their reported traditional uses. We found that most of the medicinal uses of *Mercurialis* spp. are supported by scientific evidence. This includes its antidiabetic and antihypertensive properties attributable to the flavonoid rutin and narcissin, respectively; its benefits in the treatment of skin dark spots, attributable to mequinol; and its anti-inflammatory activity, attributable to scopoletin, kaempferol, squalene, and cycloartenol. This review contributes to the validation of the medicinal uses of *Mercurialis* spp. in Spain and provides some new avenues for further investigations on the biological activity of this interesting medicinal plant.

## 1. Introduction

The genus *Mercurialis* L. belongs to the Euphorbiaceae family. In Spain, it is represented by seven species (*Mercurialis annua* L., *Mercurialis ambigua* L., *Mercurialis perennis* L., *Mercurialis huetii Hanry*, *Mercurialis tomentosa* L., *Mercurialis elliptica* Poir., and *Mercurialis reverchonii* Rouy) [1]. The most widespread species are *M. annua*, *M. ambigua*, *M. perennis*, and *M. tomentosa*. The distribution of other species are being reduced to endemic areas [2].

*Mercurialis* spp. have been used since ancient times in traditional Spanish medicine. For example, in the Middle Ages, infusions made from *Mercurialis* spp. aerial parts were used to treat constipation, bronchitis, rheumatism, gout, and gynecological disorders, as well as to relieve swellings [3,4]. Today, *Mercurialis-*based remedies are used for inflammation, poor healing wounds, and sores. In Spain, the traditional uses of *Mercurialis* spp. include the treatment of high blood pressure, high blood sugar levels, and skin pigmentation disorders [5]. The fresh parts of *Mercurialis* are poisonous and their consumption has been reported to cause nausea, vomiting, and hemorrhagic inflammation of the digestive system and of the kidneys in mammals [6,7]. 

In 2014, the Spanish Ministry of Environment took the initiative of compiling all traditional knowledge relative to biodiversity, under Phase I from the inventory following the 42/2007 Law. The species *M. annua, M. ambigua, M. perennis*, and *M. tomentosa* were included in the Phase II of this inventory published in 2018 [8,9]. 

Several studies characterizing the chemical composition of *Mercurialis* spp. have already been published [5,10,11,12,13,14,15,16,17,18,19]. Here, we reviewed the chemical composition of the four aforementioned species in order to elucidate which phytochemicals could be the basis of the claimed therapeutic activity of *Mercurialis* in Spanish folklore. 

## 2. Results

### 2.1. Mercurialis *spp.*: Botanical Description, Chorology, and Variability

**Figure 1:**
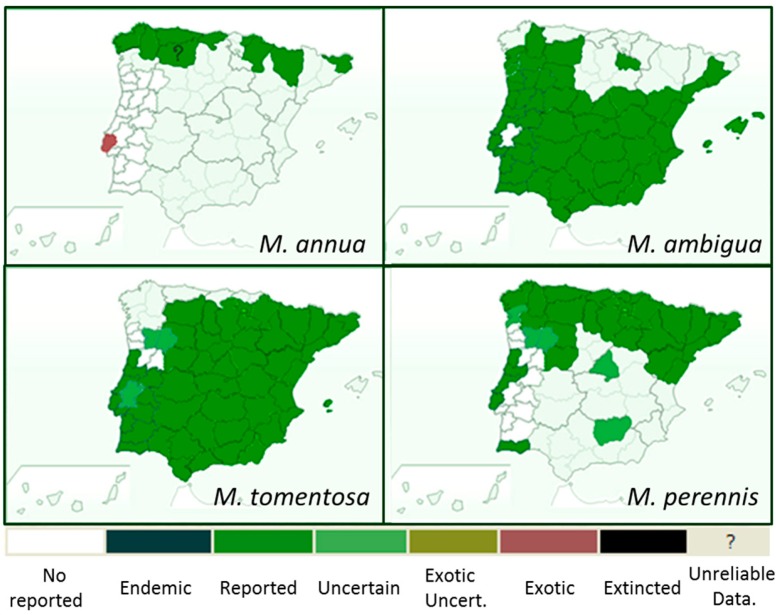
Distribution of *Mercurialis annua*, *Mercurialis ambigua*, *Mercurialis tomentosa*, and *Mercurialis perennis* in the Iberian Peninsula (https://www.bioscripts.net/).

*Mercurialis* is a genus of herbaceous plants belonging to the Euphorbiaceae family that grow in ruderal lands and grow less so in forests. In Spain, seven species have been described, most of them with a limited distribution, except for the most widespread *M. annua*/*M. ambigua* (considered the same plant by popular knowledge, and difficult to differentiate from a taxonomical point of view [1]), *M. tomentosa* and *M. perennis*, whose distribution is shown in Figure 1. *M. annua* and *M. ambigua* are annual herbs which grow in urban areas or modified agricultural fields. *M. tomentosa* is a shrub, frequent at the edges of routes with calcareous soils. In contrast, *M. perennis* is a perennial herb that has a perennial rhizome system and is primarily found in shady woods (mainly oak and beech) [1].

*Mercurialis* spp. are dioecious, rhizomatous to annual herbs. They can be glabrous or densely hairy. The quadrangular stalk has simple leaves, bistipulate and opposite on a short petiole. They can reach a length up to 40 cm. Unisexual flowers grow in different plants (dioecious species). Male spikes arise from the axils of the middle and upper leaves, the inflorescence generally extending beyond the leaves. Male flowers sessile or shortly stalked on pedicels 1–2 mm long, 4–5 mm across, consisting of three ovate–acuminate, concave, green glabrous segments, and 8–28 stamens. Female inflorescence is also axillary, shorter than the male, and usually bear 2–6 flowers, each of them with three sepals and one pistil. Hirsute, oval fruits, 5 mm in size [1].

### 2.2. Mercurialis *spp.*: Ethnobotanical Uses

*Mercurialis* spp. have been widely used in traditional Spanish medicine, despite being considered toxic plants for humans and animals [20,21,22]. A summary of the medicinal uses of *Mercurialis* spp. is shown in Table 1. 

In their Dictionary of Natural History in the Canary Islands, Viera and Clavijo reported on the use of *M. annua* as a laxative [23]. Its juice was also widely used as a purgative in the Spanish steppe areas [24]. The laxative activity of *Mercurialis* has been reported for the four species addressed in this review. For example, in Cataluña, balls of crushed leaves from *M. annua/M. ambigua*, mixed with olive oil, were used as suppositories in case of constipation [25]. A decoction of the dried [26] or fresh leaves [25,27,28,29] of *M. annua/M. ambigua* was also used for constipation in Huesca [26], Gerona [25,28,29], and Castellón [27]. In Valencia, the dried leaves were reported to be less effective than the fresh ones [30]. The blossomed stems of *M. tomentosa* were also traditionally used for this purpose in Albacete (Castilla la Mancha) [31,32]. They were also reported in the same region as an effective remedy for diarrhea [32]. In Valencia, an infusion of the aerial parts was traditionally used as a laxative, and in Castellon, it was used as a strong laxative when mixed with *Cassia angustifolia* [27]. In Valencia, the intake of an infusion with honey at fast every morning was said to act as a mild laxative [30].

An infusion of the blossomed aerial parts from *M. tomentosa* was reported to decrease blood pressure in Castilla la Mancha [31,32,37,38] and Valencia [27]. The same effect has been stated for the other *Mercurialis* species investigated in this work, but the way the plant was processed is unknown [20]. Another important medicinal use of *M. annua*/*M. ambigua* and *M. perennis*, commonly employed in the Canary Islands, was to decrease blood sugar levels [20].

The use of the four *Mercurialis* species to treat diseases associated with the respiratory system has been reported throughout Spain. For example, in the Canary Islands, they were used to treat sore throats and lung diseases [35], while in Valencia, cold and bronchitis symptoms were ameliorated with *M. tomentosa* [40,41].

An interesting use of *M. annua*/*M. ambigua* practiced in Navarra was to reduce dark spots on the skin with the application over the affected areas of the fresh parts of the plants macerated in olive oil blended with bee wax [36].

The historical use of *Mercurialis* spp. to treat gynecological disorders has also been reflected in the Spanish ethnobotanical knowledge. For example, *M. tomentosa* has been used for this aim in Valencia [40]. *M. annua*/*M. ambigua* have been used to induce miscarriage in pregnant women in Cataluña [21] and to suppress lactation in the Canary Islands [34].

Finally, the anti-inflammatory activity of *M. tomentosa* in Castilla la Mancha has been reported in different studies [31,32] and could explain the use of this species in the treatment of rheumatism and swellings [40], contusions [31,38], articular pain [42], and broken bones [31,38], as well as wound healing [32]. In most of the cases, except for broken bones, the decoction of *Mercurialis*, alone or in combination with some other plants, was used topically.

### 2.3. Towards a Validation of the Pharmacological Effects of Mercurialis *spp.*

**Figure 2:**
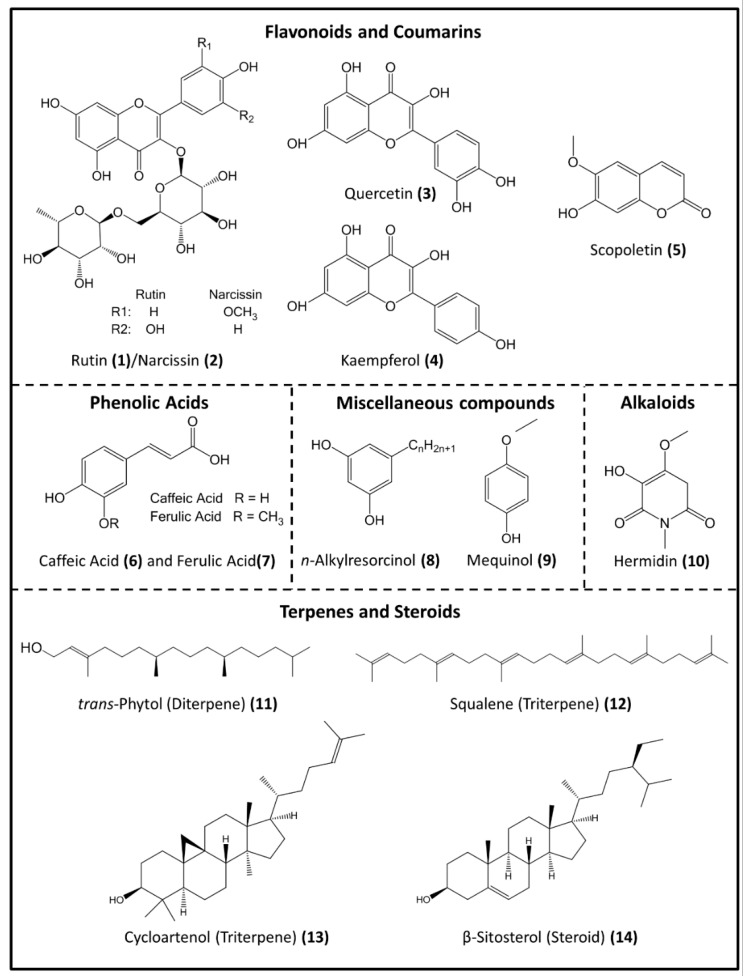
Chemical structures of the main bioactive phytochemicals in *Mercurialis* spp. (from PubChem [44]).

The main phytochemicals present in *Mercurialis* spp. are presented in Figure 2. They are flavonoids, coumarins, alkaloids, phenolic acids, terpenes and steroids, and miscellaneous compounds, such as simple aromatic constituents and phenolic lipids [5,14,15,19,43].

### 2.4. Experimental Studies on Activity 

The chemical composition of *Mercurialis* spp. and the biological activity of the phytochemicals that could explain the traditional medicinal uses of this plant in Spain are presented in Table 2.

Among the compounds compiled in Table 2, the potential of the flavonoids rutin (**1**) and narcissin (**2**) to decrease blood sugar levels [45,46,47] and blood pressure [48], respectively, are especially remarkable. Another well-demonstrated effect is the use of mequinol (**9**) to treat skin depigmentation [49,50,51], compatible with the use of *M. annua/M. ambigua* described in Navarra as a skin whitening agent [36].

### 2.5. In Silico Modelling for Hermidin Target Prediction 

Since no scientifically proven biological activities were found for the alkaloid hermidin, *in silico* modelling was done using the software Swiss Target Prediction [76]. Computational predictions of bioactive molecule targets based on similarity with known ligands were done to narrow down the number of potential targets and rationalize side effect of molecules. The main results obtained for *Homo sapiens* are shown in Table 3. From all the targets analyzed, the membrane muscarinic acetylcholine receptors (mAChRs) M1, M2, M3, M4, and M5 showed the highest probability to be hermidin targets. Similar results were obtained for *Mus musculus* and *Ratus norvegicus* (data not shown). The mAChRs antagonist action shown for other well-known alkaloids, such as scopolamine and atropine [77], support the result of our computational modelling.

## 3. Discussion

Despite their toxicity, *Mercurialis* spp. have been widely used medicinally since ancient times, although they are relatively underused nowadays. When carrying out this review on the traditional knowledge concerning four *Mercurialis* species (*Mercurialis annua* L., *Mercurialis ambigua* L., *Mercurialis perennis* L., and *Mercurialis tomentosa* L.) in Spain, we found a great variety of medicinal uses.

Several studies have been published on the chemical composition of *M. annua* [15,43,78], *M. perennis* [5,10,12,14,15,16,79,80], and *M. tomentosa* [19], confirming the presence of a wide variety of phytochemicals in these species (Table S1). Some of them possess some interesting biological activity, which has already been extensively validated scientifically (Table 2). Previous studies on the medicinal uses of *Mercurialis* spp. and their constituents are detailed below according to distinct body systems.

### 3.1. Circulatory System 

*M. annua/M. perennis* have been used to control blood sugar levels, an effect that can be attributed to the presence of quercetin-3-*O*-rutinoside (rutin) (**3**) and ferulic acid (**7**). The antihyperglycemic properties of rutin (**1**) have been widely demonstrated, as well as its potential to prevent and treat pathologies associated with diabetes, nephropathy, neuropathy, liver damage, and cardiovascular disorders. The mechanisms proposed for the antihyperglycemic effect of rutin (**1**) include a decrease in the absorption of carbohydrates, an inhibition of gluconeogenesis, an increase in tissue glucose uptake, a stimulation of the secretion of insulin, and a protection of the Langerhans islets against degeneration [46]. The activity of ferulic acid (**7**) in type 2 diabetes was demonstrated in high-fat and fructose-induced type 2 diabetic rats, where the blood glucose and serum insulin levels were restored, improving insulin sensitivity and hepatic glycogenesis, inhibiting gluconeogenesis and negative regulators of insulin signaling to maintain normal glucose homeostasis [67].

Another flavonoid component of *M. annua* is isorhamnetin-3-*O*-rutinoside (narcissin) (**2**), which could be responsible for the antihypertensive action of this plant, as it has previously demonstrated such an effect [48]. The flavonoid kaempferol (**4**), which can relax smooth muscles, could also explain the antihypertensive effect [61].

### 3.2. Digestive System 

The antidiarrheal effect of *Mercurialis* spp. described in Castilla la Mancha could rely on the spasmolytic action of the flavonoids rutin (**1**) and quercetin (**3**) and of the coumarin scopoletin (**5**) [52,62]. On the other hand, the extensive use of *Mercurialis* spp. as laxatives could be explained by the antagonistic actions obtained in some cases by the same plant. An example of this has been reported in a recent publication in which the spasmolytic and spasmogenic activity of *Punica granatum* Linn. were described with high and low doses of plant extracts, respectively [81]. The spasmogenic response seems to be triggered by cholinergic activation, whereas the spasmolytic effect appears to be mediated by the blockade of voltage gated calcium channels. The molecule responsible for the spasmogenic action could be caffeic acid (**6**), a phenolic acid also present in peach leaves, which also have spasmogenic activity [82].

The cholagogue/anticholagogue action reported for *M. tomentosa* in Valencia is another example of how antagonistic effects can be observed for the same plant. Ferulic acid (**7**) is categorized as a cholagogue and choleretic compound by the MeSH (Medical Subject Headings) from PubMed [68]. No scientific evidence supporting the anticholagogue effect of any of the phytochemicals from *Mercurialis* spp. has been found.

The hepatoprotective activity of the blossomed stems of *M. tomentosa* reported in Castilla la Mancha and Murcia could rely on scopoletin (**5**), only present in this species so far [19]. A recent study has shown a reduction in the level of hepatic lipid accumulation and liver inflammation in diabetic rats treated with scopoletin to the same extent as the antidiabetic drug metformin [65]. In spite of the absence of reports on *M. annua/M. ambigua/M. perennis* in folk knowledge concerning its liver protective action, the presence of squalene (**12**) in *M. perennis* could make this plant a good candidate to treat hepatic disorders, since it has been demonstrated that squalene (**12**) could accumulate in the liver decreasing the levels of hepatic cholesterol and triglycerides [73]. This could have a beneficial effect on the gallbladder, due to the fact that low cholesterol hepatic levels are necessary to reduce the formation of gallstones [83].

### 3.3. Skin 

The treatment of dark spots on the skin using *M. annua*, as reported in Navarra, could be attributed to the presence of 4-methoxyphenol (mequinol or 4-hydroxyanisole-4-HA-) (**9**). The latter is a common topical treatment for melasma, commercialized at a concentration of 2% in combination with 0.01% tretinoin [49]. It acts as a substrate for tyrosinase and inhibits the formation of melanin precursors [49]. Its role as a tyrosinase substrate also makes 4-HA (**9**) a promising agent to treat melanoma [84], since 4-HA (**9**) can be oxidized to 4-methoxycatechol and its o-quinone [84]. The latter is a potent electrophile agent which binds covalently to protein thiols and/or glutathione, leading to melanoma cell death [85]. 4-HA (**9**) is also used topically to treat depigmentation in patients with vitíligo when extensive body surface areas are unresponsive to repigmentation therapies [51]. 

### 3.4. Respiratory System 

The activity of *M. perennis* against lung infections may be attributed to the sterol β-sitosterol (**14**). Indeed, it has been demonstrated that the interaction between β-sitosterol (**14**) and pneumolysin, a virulence factor from *Streptococcus pneumoniae*, affects the ability of *S. pneumoniae* to colonize the lungs [75]. 

### 3.5. Urinary and Genital Systems

*Mercurialis* spp. are used traditionally to treat gynecological disorders. A recent study has demonstrated the role of *trans*-phytol (**11**), a diterpene present in *M. perennis*, as an aromatase inhibitor. Aromatase is an enzyme involved in the biosynthesis of estrogens, and aromatase inhibitors are a way to prevent or treat estrogen-mediated carcinogenesis, including breast, ovarian, and endometrial cancers [86]. The presence of *trans*-phytol (**11**) in the aerial parts of *M. perennis* suggests that this plant could be important in the prevention or treatment of estrogen-dependent carcinogenesis [71]. 

The inhibition of estrogens biosynthesis by *trans*-phytol (**11**) could also explain the use of *M. perennis* as an abortive agent, since estrogens are required for the continuation of pregnancy. Besides *trans*-phytol (**11**), the aforementioned spasmogenic action of caffeic acid (**6**) could also play a determinant role in the abortive action of *M. perennis*, since it could be involved in triggering uterine contractions needed for the termination of pregnancy. The relation between plants with spasmogenic properties and miscarriage being a well-established feature [87]. Although the spasmogenic action of caffeic acid (**6**) has not been clearly demonstrated, a previous study showed that another phenolic acid (gallic acid) had a spasmogenic effect [87]. 

The treatment of urinary and gynecological conditions, such as renal colic and dysmenorrhea reported in Valencia, could also rely on the antispasmodic action of rutin (**1**), quercetin (**3**), and scopoletin (**5**) [88,89].

The relief of menstrual molimina symptoms could rely on the presence of β-sitosterol (**14**) (the most abundant phytosterol in *M. perennis* -14.2%-) and campesterol (2.4%). A similar action has been demonstrated for Evening Primrose (*Oenothera biennis* L.) oil, which is widely used as a natural therapy to ease menstrual molimina symptoms. In evening primrose oil, both sterols are involved in inhibiting the release of different proinflammatory mediators [90]. 

### 3.6. Others

All *Mercurialis* spp have been used traditionally to treat arthrosis and inflammatory-mediated disorders, although the anti-inflammatory activity is more prominent in *M. tomentosa*. The latter has been employed to relieve rheumatism, swellings toothaches, and for contusions, broken bones, knee and feet pain, and wound healing. The anti-inflammatory properties of *M. tomentosa* could be explained by the presence of scopoletin (**5**) [19], which has demonstrated anti-inflammatory activity through cytokine suppression [63,64]. Other compounds with well-established antioxidant properties and present in *Mercurialis* spp. that could also contribute to the anti-inflammatory effect of this plant include kaempferol (**4**), squalene (**12**), and cycloartenol (**13**) [58,59,73,74]. Kaempferol (**4**) (a component of *M. perennis*) has already demonstrated efficacy in the treatment of arthrosis [59], which is the only anti-inflammatory use reported for *M. perennis/M. annua*. 

Finally, despite the lack of scientific evidence linking the piperidinedione alkaloid hermidin (**10**) with the medicinal uses of *Mercurialis* spp., this alkaloid deserves a special mention here. Although there were some suggestions that this compound could be associated with the plant’s toxicity, it was the alkaloid pyridine-3-carbonitrile that was identified as the toxic principle in *M. annua* [15]. Interestingly, in a study made using the Swiss Target Prediction Software [76,91] to predict any possible interaction of hermidin with cellular molecules, we observed that hermidin (**10**) showed some strong interactions with membrane muscarinic acetylcholine receptors M1, M2, M3, M4, and M5. 

The scientific validation of the traditional medicinal uses of *Mercurialis* spp. presented in this review places this genus in a promising position to suggest further scientific research. This should focus on testing its extracts and all its phytochemicals components for biological activity to provide a direct validation of its medicinal properties as described in traditional Spanish medicine. 

In summary, this review contributes to the preservation, documentation, and validation of the traditional medicinal uses of the most prominent *Mercurialis* spp. from the Iberian Peninsula and lays the foundation for new scientific investigations on this medicinal plant.

## 4. Materials and Methods 

A bibliographic search was performed to provide scientific evidence for the medicinal uses of the four *Mercurialis* species under study. The chemical composition of these plants and the biological activity of the phytochemicals described were established using the following databases: Scopus, Dialnet, Medline, PubMed, ScienceDirect, Google Patents, Google Scholar, and Wiley Online, following a Prisma 2009 flow diagram methodology [92]. 

The chemical structures of the compounds presented in this work were retrieved from PubChem [44], a database of chemical compounds, maintained by the National Center for Biotechnology Information (NCBI), a component of the National Library of Medicine of the National Institues of Health (NIH). All structures were drawn and edited using the software ChemDraw Professional 17.0 (Perkin Elmer)

The Swiss Target Prediction Software [76,91], a free webserver to accurately predict the targets of bioactive molecules based on 2D and 3D similarities with known ligands, was used to predict the probable molecular targets of hermidin. 

## Figures and Tables

**Table 1:** Traditional uses of *Mercurialis* spp. in Spanish medicine.

		Part ^1^	Formulation	Traditional Use	Location [Ref.]
***M. annua, M. ambigua, M. perennis***	Circulatory System	U	U	Anti-hypertensive	Canary Islands [20]
	Digestive System	U	U	Laxative	Canary and Balearic Islands [20,33]
		AP	Dried Leaves/Fresh AP (decoction)	Laxative	Aragon [26] Cataluña [25,28,29] Valencia [27]
		AP	Balls of crushed leaves (suppositories)	Laxative	Cataluña [25]
	Conception, pregnancy, childbirth, puerperium, breastfeeding	U	U	Abortive	Cataluña [21]
		U	Plant kept between the breasts and back or under the armpits	Lactation suppression	Canary Islands [34]
	Endocrine System	U	U	Anti-hyperglycemic	Canary Islands [20]
	Respiratory System	U	U	For sore throat and lung diseases	Canary Islands [35]
	Musculature and skeleton	AP	Fresh plant (infusion in vine tree liquor used as a rub)	Arthrosis	Canary Islands [34]
		AP	Fresh leaves		
	Dermatological	U	U	Hair loss	Canary Islands [35]
		AP	Maceration in olive oil at low temperature and mixed with bee wax for topical use	Skin whitening agent	Navarra [36]
***M. tomentosa***	Circulatory System	AP	Blossomed AP (infusion)	Anti-hypertensive	Castilla la Mancha [31,32,37,38] Valencia [27]
	Digestive System	WP	Decoction used as a mouthwash	Toothaches	Castilla la Mancha [31,32,37,38] Valencia [27]
		WP	Blossomed plant (infusion)	Hepatoprotective	Castilla la Mancha [31,32] Murcia [39]
		AP	Blossomed stems (infusion)	Cholagogue action	Valencia [30]
		U	U		Valencia [27]
		U	U	Anticholagogue	Valencia [40]
		AP	Blossomed stems (infusion)	Intestinal infections	Valencia [30]
		AP	Blossomed stems	Diarrhoea	Castilla la Mancha [32]
				Constipation	Castilla la Mancha [31]
		U	U	Laxative	Valencia [41] Murcia [39]
		AP	Overnight infusion (mixed with *Cassia angustifolia* and taken in the morning	Strong laxative	Valencia [27]
		AP	Infused taken in the morning with honey	Mild laxative	Valencia [30]
	Urinary and Genital Systems	AP	U	Urinary and gynecological conditions	Valencia [40]
		U	Mixed with *Equisetum* spp.	Kidney swelling	Valencia [41]
	Respiratory System	U	U	Cold and bronchitis	Valencia [41]
		AP	U	Cold	Valencia [40]
	Musculature and skeleton	U	U	Rheumatism and swelling	Valencia [40]
		WP	WP decoction applied topically on a damp cloth	Contusions	Castilla la Mancha [31,38]
				Swelling legs	Andalucia [42]
		WP	Mashed plant used as a plaster	Broken bones	Castilla la Mancha [31,38]
		AP	Blossomed AP (decoction with salt and vinegar kept for 7 days in darkness)	Calcaneal spur	Castilla la Mancha [31]
		U	Decoction (with other plants such as thyme, rosemary, bay or pita)	Knee and feet pain	Andalucia [42]
	Skin and subcutaneouscellular tissues	AP	Decoction used topically	Hand cracks	Andalucia [42]
				Wound healing	Castilla la Mancha [32]
	Other	AP	U	Anti-inflammatory	Castilla la Mancha [31,32]

^1^ Part used: U, unknown; AP, Aerial part; R, Root; WP, whole plant.

**Table 2:** Biological activity of selected phytochemicals from *Mercurialis* spp.

Chemical Class	Phytochemicals	Biological Activity	Reference
Flavonoids	Rutin (**1**)	Spasmolytic	[52]
		Anti-hyperglycemic	[45,46,47]
		Anti-oxidant	[47,53]
	Narcissin (**2**)	Anti-hypertensive	[48]
	Quercetin (**3**)	Spasmolytic	[54,55]
		Antidiarrhoeal	[56]
		Anti-hypertensive	[57]
	Kaempferol (**4**)	Anti-oxidant	[58]
		Anti-inflammatory	[59]
		Anticancer	[60]
		Spasmolytic	[61]
Coumarins	Scopoletin (**5**)	Spasmolytic	[62]
		Anti-inflammatory	[63,64]
		Hepatoprotective	[65]
Phenolic acid	Caffeic Acid (**6**)	Anti-oxidant	[66]
	Ferulic acid (**7**)	Antidiabetic	[67]
		Cholagogue and Choleretic	[68]
Phenolic	n-Alkylresorcinol (**8**)	Muscle atrophy prevention	[69]
Lipids		Anti-obesity and glucose intolerance suppression	[70]
Simple Phenolics	4-Methoxyphenol (**9**)	Skin Depigmentation	[49,50,51]
Alkaloids	Hermidin (**10**)	Not described	-
Diterpenes	*trans*-Phytol (**11**)	Aromatase Inhibitor Treatment of estrogen-dependent cancer	[71]
Triterpenes	Squalene (**12**)	Anti-hypercholesterolemic	[72,73]
		Antihypertriglyceridemic	[73]
		Anti-oxidant	[73]
		Anti-inflammatory	[73]
Steroids	Cycloartenol (**13**)	Anti-inflammatory	[74]
		Antitumor	
		Anti-oxidant	
	β-Sitosterol (**14**)	Prevention of *Streptococcus pneumoniae* (lung) infection	[75]

**Table 3:** Hermidin targets obtained by computational modelling using Swiss Target Prediction Software.

Target	Common Name	Uniprot ID	ChEMBL ID	Probability*	#sim. Cmpds (3d/2D)	Target Class
Muscarinic acetylcholine receptor M1	CHRM1	P11229	CHEMBL216	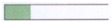	1/2	Membrane receptor
Muscarinic acetylcholine receptor M2	CHRM2	P08172	CHEMBL211	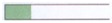	1/1	Membrane receptor
Muscarinic acetylcholine receptor M3	CHRM3	P20309	CHEMBL245	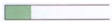	1/2	Membrane receptor
Muscarinic acetylcholine receptor M4	CHRM4	P08174	CHEMBL1821	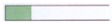	1/2	Membrane receptor
Muscarinic acetylcholine receptor M5	CHRM5	P08912	CHEMBL2035	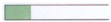	1/2	Membrane receptor

## Supplementary Materials

The following are available online at https://www.mdpi.com/2223-7747/8/7/193/s1, Table S1: Chemical composition of *Mercurialis* spp.
