# Supplementary material for: Bioactive Phytochemicals from Mercurialis spp. Used in Traditional Spanish Medicine
Source: Plants (Basel). 2019 Jun 28;8(7):193. doi: 10.3390/plants8070193 (PMC6681364; doi:10.3390/plants8070193)
Supplement: Supplementary file 1 [file plants-08-00193-s001.pdf]

M. annua	*M.perennis*	*M.tomentosa*	MOLECULE	CHEMICAL GROUP
[1]			Quercetin-3-*O*-rutinoside (Rutin)	FLAVONOIDS
[1]			Isorhamnetin-3- *O*-rutinoside (Narcissin)	
[1]			Quercetin-3-*O*-(2G-glucosyl)-rutinoside	
[1]			Isorhamnetin-3-*O*-rutinoside-4′-*O*-glucoside	
[1]			Isorhamnetin-3-*O*-rutinoside-7-*O*-glucoside	
	[2]		Quercetin	
	[2]		Kaempferol	
		[3]	Scopoletin	
[4]			Pyridine-3-carbonitrile	ALKALOIDS
[4]			Nicotinamide	
[4]	[4,5]	[3]	Hermidin	
[4]	[4,5]	[3]	Hermidin quinone	
[4]	[4,5]		*cis*-myrtanal	CARBALDEHIDE
	[5]		(-)-*trans*-myrtanol (trans-MyrOH)	TERPENES
[4]	[4,5]		*cis*-myrtanol	
	[5]		*trans*-phytol	
	[5]		Squalene	
	[5]		β-carotene	
	[5]		Phytadiene isomers	
	[5]		Nonacosane	ALKANE
	[5]		4-Methoxyphenol (Mequinol, 4-Hydroxyanisole)	AROMATIC ALCOHOLS
	[5]		3,4-Dimethoxyphenol	
		[3]	4-Hydroxybenzaldehyde	
		[3]	4-Hydroxybenzonitrile	
		[3]	4-Hydroxyacetophenone	
		[3]	Ethyl 4-hydroxybenzoate	
		[3]	Ethyl (E)-2-hydroxycinnamate	
		[3]	Methyl (E)-4-hydroxycinnamate	
		[3]	Ethyl (E)-4-hydroxycinnamate	
		[3]	Sinapyl alcohol	
[4]	[4,5]		Benzyl Alcohol	
[4]	[4,5]		2-Phenylethyl alcohol (2-Phenylethanol)	
[4]	[4]		N-ethylaniline	

[4]	[4]		6,6-dimethylbicyclo[3.1.1]heptan-2-one (β-pinone, nopinone)	
[4]	[2,4,5]	[3]	*n*-Alkylresorcinols	**PHENOLIC LIPIDS**
	[5]		α,γ, δ-Tocopherol	**TOCOPHEROLS**
		[3]	Coniferyl alcohol	**PHENYLPROPANOIDS**
	[5]		Campesterol	**STEROIDS**
	[5]		Stigmasterol	
	[5]		β-sitosterol	
	[5]		Cycloartenol	
	[5]	[3]	α-Linolenic acid	**FATTY ACIDS**
	[5]		Linoleic acid	
	[5]		*cis*-Oleic acid	
	[2,5]	[3]	Palmitic acid	
	[5]		Stearic acid	
	[5]		Linolenic acid ethyl ester (Linolen-E)	
	[5]		*trans*-Oleic acid	
[4]	[2,4]		Phaselic acid (2-*O*-(E)-caffeoyl malic acid)	**DEPSIDES**
	[2,4]		Mercurialis acid ((-)-(2R)-2-[(E)-caffeoyloxy]-glutaric acid)	
	[2]		*p*-Coumaric acid	**PHENOLIC ACIDS**
	[2]		Ferulic acid	
	[2]		Caffeic acid	
		[3]	Vanillic acid	
	[2]		Caffeoyl glucarate	**SUGAR ACIDS**
	[2]		*p*-Coumaroyl glucarate	
	[2]		Feruloyl glucarate	
	[2]		Glucaric acid	
	[2]		Glyceric acid	
	[2]		Malonic acid	**DICARBOXYLIC ACIDS**
	[2]		Succinic acid	
	[2]		Fumaric acid	
	[2]		Malic acid	
	[2]		2-Hydroxyglutaric acid	
	[2]		Citric acid	**ORGANIC ACID**
	[2]		Arabinoic acid-1,4-lactone	**LACTONE**

Bibliography

1. Aquino, R.; Behar, I.; D'agostino, M.; De. Simone, F.; Schettino, O.; Pizza, C. Phytochemical investigation on Mercurialis annua. *Biochem. Syst. Ecol.* **1987**, *15*, 667–669.
2. Lorenz, P.; Stintzing, F.C.; Meyer, U.; Conrad, J.; Berger, M.; Duckstein, S.; Bertrams, J. Investigations into the Phenolic Constituents of Dog's Mercury (Mercurialis perennis L.) by LC-MS/MS and GC-MS analyses. *Phytochem. Anal.* **2011**, *23*, 60–71.
3. Lorenz, P.; Heinrich, M.; Conrad, J.; Heller, A.; Stintzing, F.C.; Kammerer, D.R. Comprehensive Characterisation of n-Alkylresorcinols and Other Lipid Constituents of Mercurialis tomentosa L. from Alicante, Spain. *Chem. Biodivers.* **2017**, *14*.

4. Lorenz, P.; Duckstein, S.; Conrad, J.; Knödler, M.; Meyer, U.; Stintzing, F.C. An approach to the chemotaxonomic differentiation of two European Dog's mercury species: Mercurialis annua L. and M. perennis L. *Chem. Biodivers.* **2012**, *9*, 282–297.
5. Lorenz, P.; Hradecky, M.; Berger, M.; Bertrams, J.; Meyer, U.; Stintzing, F.C. Lipophilic constituents from aerial and root parts of Mercurialis perennis L. *Phytochem. Anal.* **2010**, *21*, 234–245.
